# Financial Literacy and Economic Attitudes as Protective Factors Against Pathological Gambling? A Systematic Review

**DOI:** 10.1007/s10899-025-10375-1

**Published:** 2025-04-28

**Authors:** Chiara Barone, Guendalina Graffigna

**Affiliations:** 1 Department of Psychology, Università Cattolica Del Sacro Cuore, Largo Agostino Gemelli 1, 20123 Milan, Italy; 2 EngageMinds HUB – Consumer, Food and Health Engagement Research Center, Università Cattolica del Sacro Cuore, 20123 Milan, Italy; 3 Faculty of Agriculture, Food and Environmental Sciences, Università Cattolica del Sacro Cuore, Via Bissolati, 74, 26100 Cremona, Italy

**Keywords:** Gambling addiction, Financial Literacy, Prevention, Consumer education

## Abstract

Pathological gambling poses significant global issues, leading to economic, social, and psychological consequences such as debt, family breakdowns, and mental health problems. While various risk factors for gambling addiction, including comorbid addictions, psychiatric disorders, gender, age, and easy access to gambling venues, have been well-studied, less emphasis is placed on protective factors. Strong social support and higher education are key in mitigating gambling addiction. Higher education, in particular, equips individuals with better decision-making skills and risk management strategies, reducing the likelihood of addictive behaviors. Strengthening education and social support systems is essential for preventing gambling addiction. A systematic review was conducted across Scopus, PubMed, Web of Science, and EBSCO, focusing on studies published after 2000. Peer-reviewed studies written in English that examined the relationship between financial literacy and gambling were included. Studies focusing solely on financial topics or not in English were excluded. The review follows the PROSPERO protocol. Financial literacy is linked to lower rates of pathological gambling, although its impact varies based on cultural context and gambling accessibility. From 880 papers, 8 met the inclusion criteria. Six studies confirmed a relationship between higher financial literacy and reduced gambling behavior, while two studies indicated that the significance of this relationship depended on specific financial literacy dimensions or contextual factors. This research underscores the importance of incorporating consumer education and psychological factors into future gambling addiction prevention strategies, particularly for younger gamblers.

## Introduction

### Background

Pathological gambling represents a significant global problem with devastating impacts on the lives of individuals and their families (Armoon, et al., 2023). Recent epidemiological data (Gabellini, et al., 2022) highlight the significant global impact of gambling. In 2022, the U.S. gambling industry achieved a record-breaking revenue of over $60 billion, largely fueled by in-person gambling, which accounted for more than 80% of the industry's total earnings. Online gambling, which has shown consistent growth, now represents almost 20% of global gambling activities. Similarly, Europe’s gambling market saw substantial growth, with gross gaming revenue increasing by 23% in 2022, reaching €108.5 billion. Online gambling played a key role in this surge, contributing an 8% increase and generating €38.2 billion. Public health concerns are escalating due to the high rates of problem gambling, especially in regions like Australia and Europe. This phenomenon can lead to severe economic, social, and psychological consequences, including debt, family relationship breakdowns, and mental health issues (George & Murali, 2005). In literature, several studies have analyzed the relationship between gambling and other factors such as comorbidity with other addictions (Ford & Håkansson, 2020) or psychiatric disorders (Lorains, et al., 2011), gender (Moore & Grubbs, 2021), age (Pettorruso et al., 2021), family experiences with gambling (Dowling et al.), genetic predisposition (Solé-Morata et al., 2023), and ease of access to gambling venues (Ofori et al., 2020). These have been identified as possible risk factors for pathological gambling. However, there is less information regarding protective factors, which are equally important in mitigating gambling addiction. Risk factors are defined as antecedent conditions that increase the likelihood, severity, and duration of problem gambling (Coie et al., 1993; Farrington and Ttofi, 2011; Kazdin et al., 1997; Kraemer et al., 1997). Conversely, protective factors are conceptualized as antecedent conditions that reduce the likelihood of problem gambling onset, regardless of exposure to identified risk factors (Coie et al., 1993; Farrington and Ttofi, 2011; Kazdin et al., 1997; Kraemer et al., 1997; Lussier et al., 2014; Shead et al., 2010) (Dowling et al., 2021a, 2021b). For example, recent literature has shown the importance of the parental role as a preventive factor for adolescents (Dowling et al., 2021a, 2021b). Social support and social bonds are identified as protective factors against gambling problems (Dowling et al., 2017a, 2017b). The emotional support, personal growth, and autonomy, along with overall well-being, mitigated the effects of gambling motives and high-risk situations, indicating that these factors served as protective elements (Dowling N. A., Aarsman et al., 2021). Conversely, the perception of insufficient social support from loved ones is a recognized risk factor for the development and persistence of gambling problems (Hardoon et al., 2004; Petry & Weiss, 2009). At-risk or problem gamblers tend to report lower perceived social support (Canale et al., 2017). Literature suggests that risk perception (Donati, et al., 2021) and education can mitigate and prevent addiction. This is why mitigation campaigns among young adults often focus on raising awareness of the harmful consequences of gambling (Taylor and Hillyard, P., 2008). Regarding education, higher levels of education are associated with lower rates of substance abuse and addiction. This correlation can be attributed to several factors. For instance, increased levels of awareness and knowledge. More specifically, individuals with higher education levels tend to have better access to information about the risks and consequences of substance abuse, which can lead to more informed decisions regarding different forms of risky behaviors (Schmengler, 2022). Another reason can be that education often leads to better job opportunities and higher income, reducing the financial stress that can contribute to substance abuse. People with higher education levels are less likely to experience the social and economic hardships that are commonly linked to addiction (Schmengler, 2022). In the context of problem gambling, it is also important to consider research that supports the specific difficulties gamblers face regarding the decision-making process and generally discusses their ability to make considered and rational decisions compared to non-gamblers or social gamblers (Clark, L., 2017). Several studies have outlined the idiosyncrasies present in the decision-making processes and risk perceptions of gamblers (Spurrier, Blaszczynski & Rhodes, 2015a, 2015b). Derevensky et al. (2010), for example, included questions in their study regarding perceived benefits, long-term risk of problems, and the likelihood of positive outcomes, finding that problem gamblers had a more optimistic view on all these aspects. Moreover, experts argue that gamblers' personal beliefs lead to an underestimation of risk and losses, poor prioritization of their needs, and inadequate planning and implementation of risk management strategies (Blaszczynski et al., 2002). Additionally, moderate to high-severity gamblers generally have lower education levels than moderate gamblers (Bastiani et al., 2013). In this context, education as a preventive factor can play a crucial role, enabling young adults to develop risk management skills and rational decision-making processes that can keep them safe from pathological gambling addiction.

### Financial Literacy

Financial literacy is defined in various ways by different scholars, but it generally encompasses the knowledge, skills, and behaviors necessary for making informed and effective financial decisions.

Financial literacy is the combination of awareness, knowledge, skills, attitudes, and behaviors necessary to make sound financial decisions and ultimately achieve personal financial well-being (OECD, 2015). According to the National Bureau of Economic Research (Kaiser & Lusardi, 2024), financial literacy includes the ability to process economic information and make decisions regarding financial planning, wealth accumulation, debt management, and retirement planning. This definition aligns with that provided by Lusardi and Mitchell, which states that financial literacy is the ability to understand and manage one's finances, including key concepts such as saving, investing, and debt management (Lusardi & Mitchell, 2014). Another definition highlights it as a critical life skill essential for achieving financial security and well-being, contributing to economic growth and sustainable development (Zaimovic, A.; Torlakovic, A. et al., 2023). In the definition of financial literacy, some divergences reflect different perspectives and approaches. The OECD (2015) adopts a broad definition that includes awareness, knowledge, skills, attitudes, and behaviors, indicating a holistic view, while Huston (2010) and Lusardi and Mitchell (2014) tend to focus more on skills and knowledge, with less emphasis on attitudes and behaviors. This observation is important because it influences the measurement approaches used to assess the level of financial education. In this paper, we support the multidimensional definition of financial literacy in line with that provided by the OECD. All approaches, however, share the general assumption that financial education is essential for making informed financial decisions and for individual and collective economic well-being. The Swiss Journal of Economics and Statistics further emphasizes that financial literacy affects saving, investment behavior, and debt management, showing that financially literate individuals are more likely to accumulate wealth and plan effectively for retirement (Lusardi A., 2019). These insights underscore the importance of financial education programs to improve financial literacy, which in turn can lead to better financial decisions, reduced financial stress, and enhanced overall economic well-being.

As previously highlighted, gambling addiction is an increasing problem with serious consequences. The literature has meticulously investigated the risk factors that can increase the likelihood of developing the addiction. It is also important to highlight the protective factors, which can indeed serve as potential safeguards against this issue. In this regard, financial literacy could significantly influence addiction behaviors. Higher financial literacy is associated with better financial decision-making and management, which can mitigate the financial stress that often leads to substance abuse (Langabeer JR et al., 2024). For instance, financially literate individuals are better at managing debt, saving, and investing, reducing the likelihood of turning to addictive behaviors as coping mechanisms (Lusardi A & Messy F.A., 2023). Furthermore, financial literacy has proven useful in the treatment of disordered money behaviors (Behfar, M. et al., 2024). Despite its importance, the potential protective effect of financial literacy against pathological gambling remains underexplored. Therefore, in this study, we assume that financial literacy programs can serve as preventive measures against gambling addiction by educating individuals on the risks and providing skills to manage finances effectively.

### Study Aim

This study explores the relationship between financial literacy and gambling behaviors, focusing on frequency and intensity. Financial literacy is crucial for managing personal finances, but its impact on gambling requires further investigation. More specifically, this paper employs a systematic review methodology to assess the existing literature on the subject and collects new data to provide deeper insights into how financial literacy might serve as a protective factor against gambling addiction.

## Material and methods

### Study selection and screening

An extensive literature review – following the PRISMA methodology (Moher et al., 2009)—was conducted to identify papers that analyze the relationship between gambling and financial education in order to gather all available information on this topic. The following search string was developed:

TITLE ( gambling OR gamblers OR gambler OR "gambling Scratch-off" OR "slot machine*" OR casino OR casinos OR bettors OR bettor OR bet OR betting OR lotteries OR lottery) AND TITLE -ABS( "financial literacy" OR "financial capability" OR "financial behavio*r*" OR "household finance" OR "personal finance" OR "financial learning" OR "money attitude" OR "economic attitude" OR "economic competence" OR "financial attitude" OR "financial knowledge" OR "financial skill" OR "management skill" OR "financial abilit*" OR "financial confidence" OR "financial literacy confidence" OR "financial outcomes" OR "financial education" OR "household finance" OR "financial confidence" OR "financial well-being" OR "financial management" OR "financial Management behavio*r" OR "financial management practices" OR "financial management attitude*" OR "financial counselling" OR "economic concept" OR "financial accuracy" OR "financial overconfidence" OR "financial confidence" OR "financial competence" OR "money management" OR "controls of spending" OR "management competence*" OR "portfolio choice*" OR "behavio*ral finance" OR "financial wellbeing" OR "money attitude*" OR "cash management" OR "risk management" OR "financial expert*" OR "risk aversion" OR "financial risk*" OR "financial perception" OR "economic behavio*r" OR "risk preference*" OR "financial decision" OR "numeracy" OR "risk management" OR "risk tolerance" OR saving* OR budgeting OR rationality OR "trading behavior" OR "financial decision making".

**Fig. 1:**
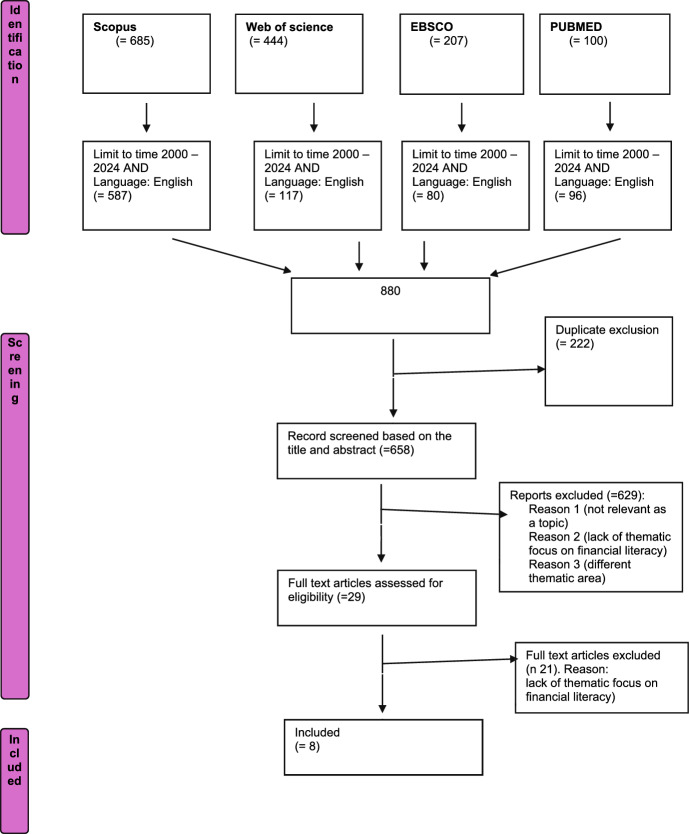
PRISMA systematic review flow diagram

Multiple terms related to finance in terms of knowledge, education, and attitude were included, given the lack of clarity regarding the definition of “financial literacy” and in line with the intent of this systematic analysis to gather consistent evidence regarding the role of financial education as a potential preventive factor for problematic gambling. The string was adapted to the requirements of various databases examined for the literature analysis: Scopus, OECD, Web of Science, PubMed, and EBSCO. No results were produced on OECD. We limited the time frame from the year 2000 to collect the most recent scientific contributions. The inclusion criteria followed are peer-reviewed, studies analyzing the relationship between financial literacy and gambling, empirical studies, studies in the English language. The exclusion criteria are studies that solely examine financial aspects but are not focused on gambling, studies not published in English. There are no exclusions based on study design; however, data not originating from scientific literature or verified sources will not be included. The research intentions were previously declared on PROSPERO (Code: 539,731). The two researchers who conducted the analyses resolved any ambiguous situations or biases by discussing the inclusion or exclusion of the articles based on the identified eligibility criteria and their expertise on the topic. Figure 1 describes the research process:

### Analysis of the studies

**Fig. 2:**
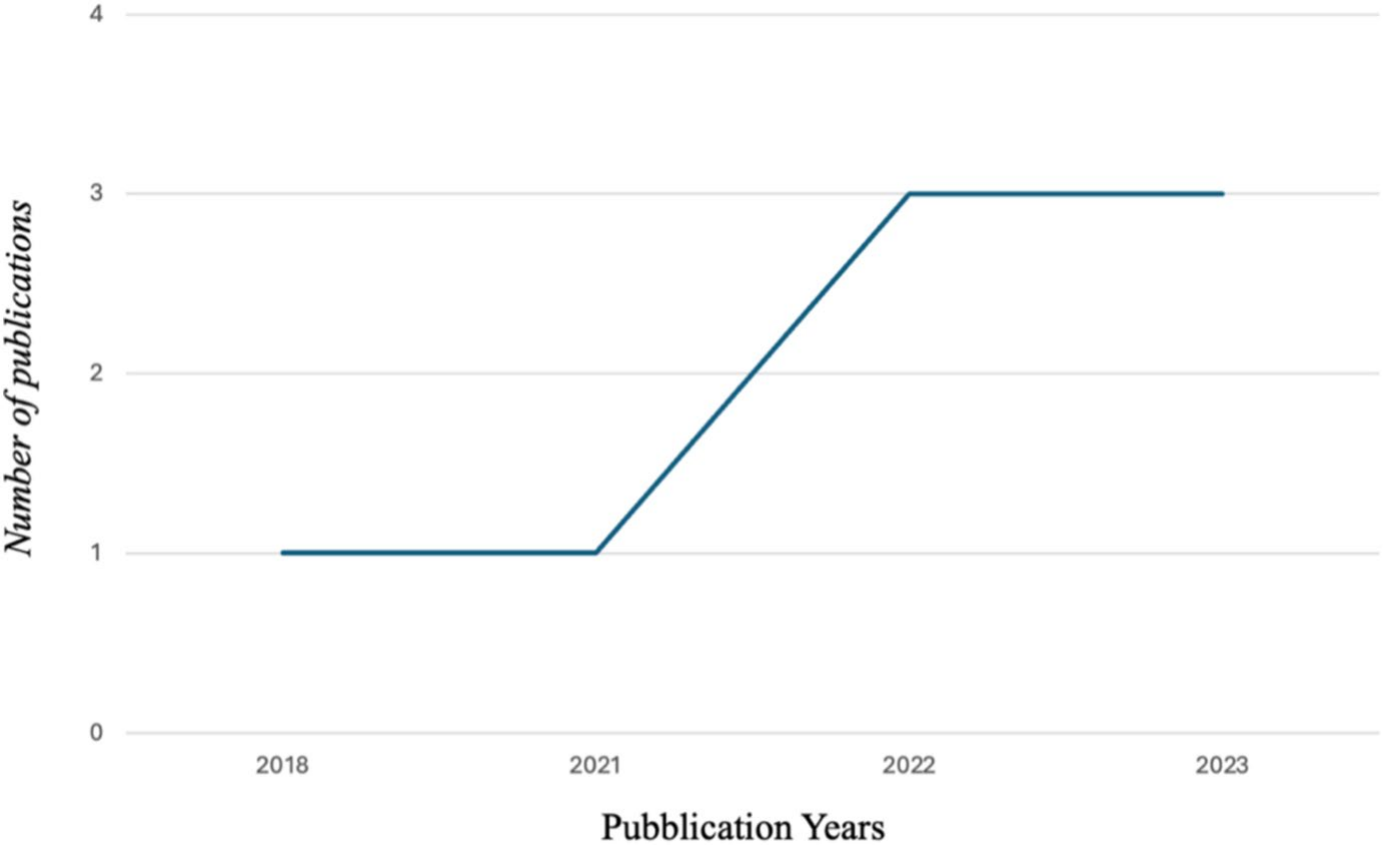
Time distribution of papers

**Fig.3:**
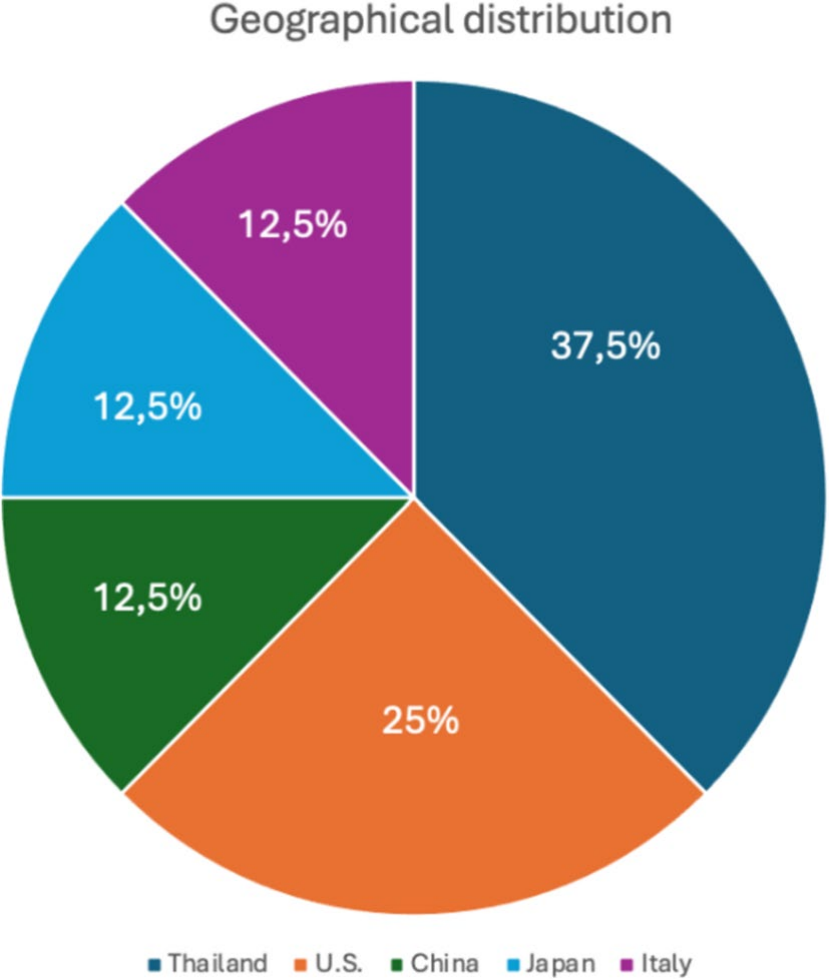
Geographical distribution of papers

**Fig.4:**
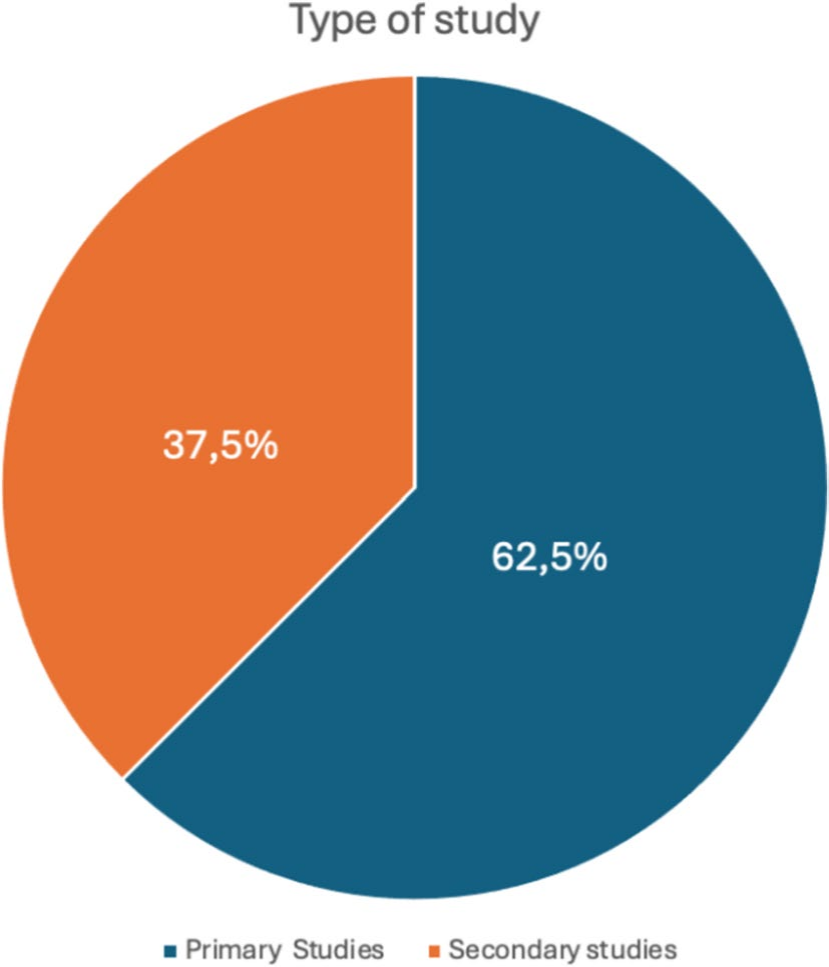
Type of study in terms of data collection

In line with the aim of the systematic review process, an initial bibliometric analysis was conducted to profile the pool of selected papers. Time trends (Fig. 2), geographical distribution (Fig. 3), and study methodologies regarding the data collection methods (Fig. 4) were thus mapped. Moreover, the first table (Table 1) lists the main characteristics of the studies, such as title, author(s), year of publication, study objective, country and the data collection methods for each article.

**Table 1:** General charchteristics of the articles

Title	Authors	Year	Aim	Country	P.S.*	S.S
Financial Literacy and Financial risk tolerance of lottery gamblers in Thailand	Amonhaemanon D	**2022**	This paper explores the effects of financial literacy levels, financial risk tolerance, and demographic characteristics on the gambling behaviors of informal laborers in Thailand	Thailand	x	
Financial Literacy Confidence and Gambling Intensity among Informal Labor	Amonhaemanon D	**2023**	This study aimed to explore whether financial literacy and financial confidence, impacts the extent of gambling intensity among informal laborers in southern Thailand	Thailand	x	
Financial stress and gambling motivation: the importance of financial literacy	Amonhaemanon D	**2023**	The primary goal was to examine the factors influencing financial stress, including the level of financial literacy and risk tolerance, among informal laborers. Additionally, the study aimed to identify the factors motivating informal workers to purchase lottery tickets, categorized by economic, psychological, and social motives	Thailand	x	
Financial literacy and its relation to lottery gambling consumption	Seungyeon C	**2022**	This study aims to investigate the relationship between financial literacy and lottery consumption frequency	U.S	x	
Financial Literacy and Gambling Behavior in the United States	Watanapongvanich S.; Binnagan P.; Putthinun P.; Saidur Rahim Khan M.; Kadoya Y	**2022**	The study investigates whether enhancing financial literacy can help decrease gambling frequency in the United States	U.S	x	
Characteristics of Chinese Lottery Consumers’ Financial						
Literacy and its Relationship with Problem Lottery Gambling	Wang Y.; Yang Z.; Xin Z	**2023**	The study aims to explore the financial literacy's level of chinese lottery consumers and to answer whether financial literacy inhibits problem lottery gambling	China	x	
Financial Literacy and Gambling Behavior: Evidence from Japan	Watanapongvanich S.; Binnagan P.; Putthinun P.; Saidur Rahim Khan M.; Kadoya Y	**2021**	This study investigates the relationship between financial literacy, financial education, and gambling behavior among the Japanese population. The authors propose that individuals who are financially literate and educated, and who apply their knowledge to make prudent financial decisions, are less likely to engage in gambling	Japan	x	
Gamblers, scratchers and their financial education	Becchetti L.; Bellucci D.; Rossetti F	**2018**	The authors developed an online survey to investigate the characteristics of slot/video poker players and individuals who purchase scratch-off lottery tickets	**Italy**	x	

^*^P.S. and S.S. indicate the type of study based on the data collection method

## Result

### Articles Overview

The final articles cover a time span from 2018 to 2023 (Fig. 2).

The search yielded a total of 880 papers. Rayyan® was used for the analysis, first removing duplicates (N = 222). The remaining papers (N = 658) were screened by title and abstract and discarded based on thematic relevance to the research objectives. Only articles addressing the relationship between financial literacy dimensions and gambling were retained.

The 29 articles were then analyzed in full text using Mendeley Reference Manager®, with 8 being included as they aligned with the objectives of our analysis. Articles were mainly excluded due to the lack of thematic relevance to our research purposes for two primary reasons: (1) articles presenting terms like “bet” and “finance” but related to the trading environment, and (2) articles not including financial literacy and its dimensions but other constructs.

Regarding the geographical distribution (Fig. 3; Table 1), three studies were conducted in Thailand (37.5%) by the same author, two others examine data related to the U.S. population (25%), one in Italy (12.5%), one in China (12.5%), and one in Japan (12.5%).

In terms of data collection for the analyses, most of the included studies (5) are primary research studies, while the remaining three used pre-existing databases (Fig. 4; Table 1). However, all data were collected through questionnaires.

### Measurements used in the Studies

It was deemed useful to represent the most commonly used measurements in the articles to assess the level of gambling problems and the level of financial literacy of the respondents.

#### Measurement of Problematic Gambling

**Fig. 5:**
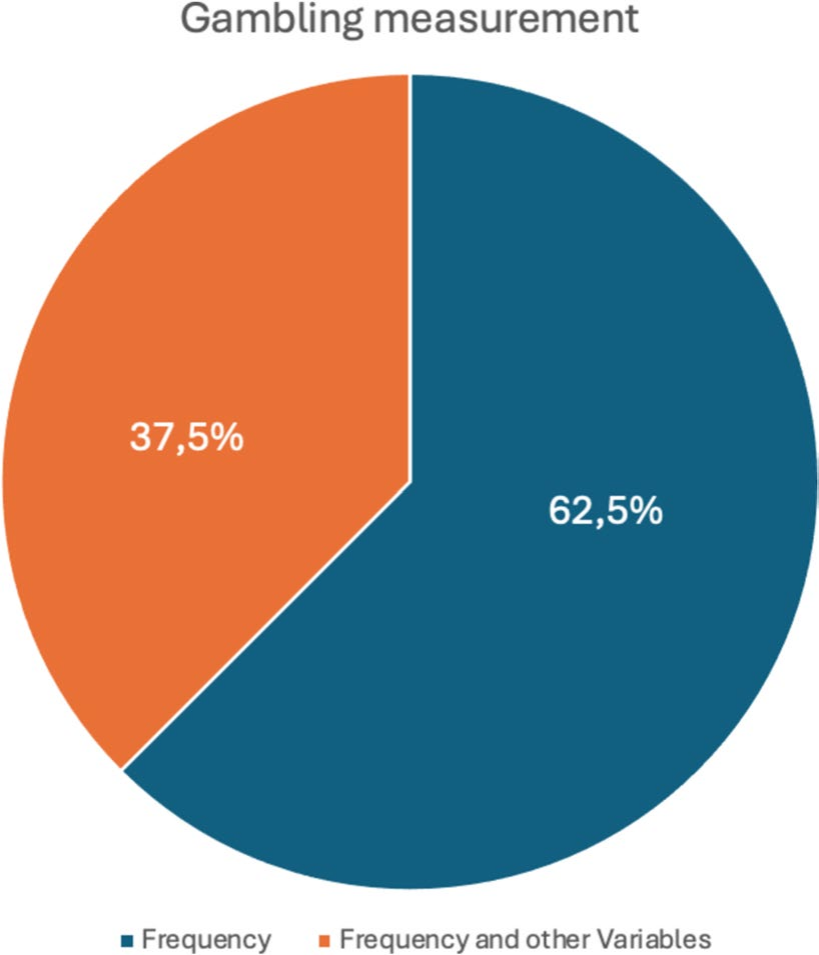
Gambling measurement

In the first case (Fig. 5; Table 2), gambling is primarily measured by its frequency. For instance, in the studies conducted by Amonhaemanon D., frequency refers to how often respondents purchase lottery tickets, which are then categorized into sub-groups based on their responses. One of the studies published by the same author also examines the amount of money bet, following the measurement concept from Pravichai and Ariyabuddhi-phongs (2015). Similarly, Seungyeon C.'s study uses the frequency of lottery ticket purchases as a variable to detect gambling problems. All the studies included assess gambling behavior based on the frequency of gambling activities, measured through one or two questions. In contrast, the study by Wang Y., Yang Z., and Xin Z. employs the scale developed by Li et al. (2012), which consists of 19 questions. This scale covers several dimensions, including: (1) harmful behavior, (2) over-expectation (5 items), which assesses whether lottery consumers have unrealistic expectations of winning, and (3) compulsive disorder (4 items), which refers to players who spend excessive time and money on lottery purchases and struggle to control their gambling. Participants responded on a 5-point scale (1 = never, 5 = always).

**Table 2:** Gambling measurement

Title	Authors	Frequency	Frequency and amount of money bet	Scale developed by Li et al	Frequency and other habits
Financial Literacy and Financial risk tolerance of lottery gamblers in Thailand	Amonhaemanon D	x			
Financial Literacy Confidence and Gambling Intensity among Informal Labor	Amonhaemanon D		x		
Financial stress and gambling motivation: the importance of financial literacy	Amonhaemanon D	x			
Financial literacy and its relation to lottery gambling consumption	Seungyeon C	x			
Financial Literacy and Gambling Behavior in the United States	Watanapongvanich S.; Binnagan P.; Putthinun P.; Saidur Rahim Khan M.; Kadoya Y	x			
Characteristics of Chinese Lottery Consumers’ Financial				x	
Literacy and its Relationship with Problem Lottery Gambling	Wang Y.; Yang Z.; Xin Z				
Financial Literacy and Gambling Behavior: Evidence from Japan	Watanapongvanich S.; Binnagan P.; Putthinun P.; Saidur Rahim Khan M.; Kadoya Y	x			
Gamblers, scratchers and their financial education	Becchetti L.; Bellucci D.; Rossetti F				x

#### Measurement of Financial Literacy

**Fig. 6:**
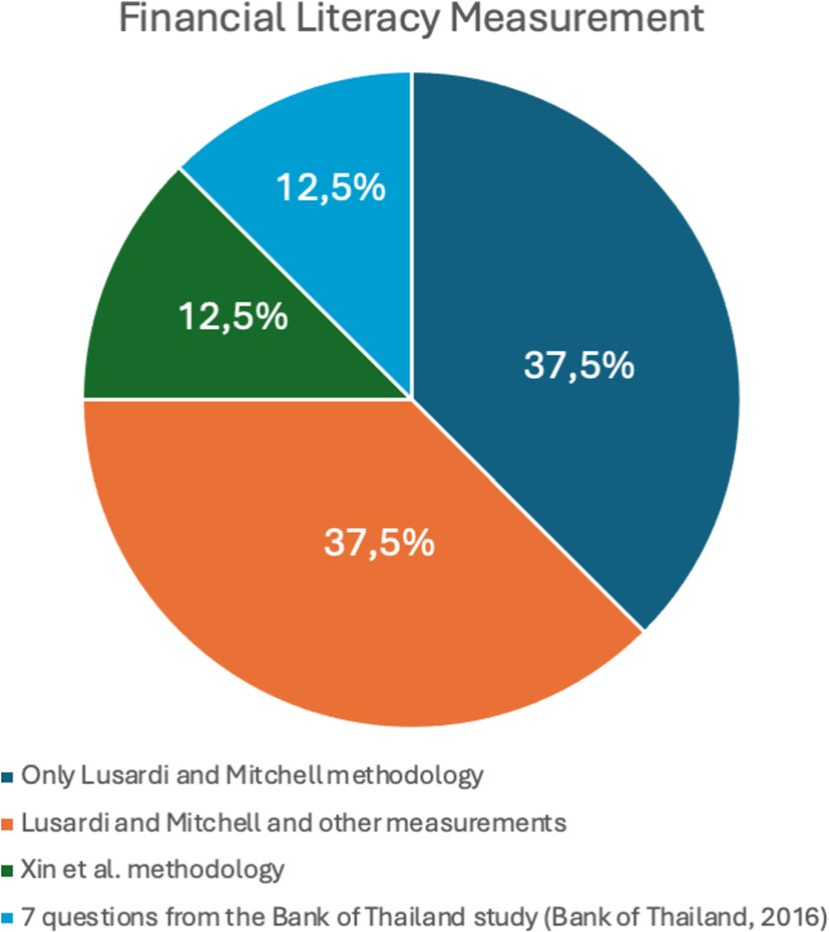
Financial Literacy measurement

Regarding the measurement of financial literacy (Fig. 6, Table 3), the most commonly used method is the one developed by economists Lusardi and Mitchell, which consists of three questions: (i) Understanding interest compounding; (ii) Understanding inflation; and (iii) Understanding risk diversification. This method was designed to assess financial literacy across various countries through a simple, concise, and relevant measurement system, which explains its broad international adoption. Another measurement method observed in our included studies, in addition to Lusardi and Mitchell's model, is the 7 questions from the Bank of Thailand study (Bank of Thailand, 2016) (Amonhaemanon D., 2022). Furthermore, the methods developed by Morgan and Trinh (2019) and the OECD/INFE (2015) (Seungyeon C, 2022.) were used alongside Lusardi and Mitchell's Big Three questions. One study (Wang Y.; Yang Z.; Xin Z., 2023) explored financial literacy using the approach by Xin et al. (2020), which introduces a new financial literacy measurement involving the index of homo economicus (average of financial knowledge, financial capacity, and financial management values) and the index of homo sociologicus (average of financial ethics and wealth values), based on the triarchic theory (Xin et al., 2023). This method includes five subtests: financial knowledge, financial capacity, financial management values, financial ethics, and wealth values.

**Table 3:** Financial Literacy measurement

Title	Authors	Three questions (Lusardi et Mitchell)	Lusardi et Mitchell and other methodologies	Xin et al	Questions from Bank of Thailand
Financial Literacy and Financial risk tolerance of lottery gamblers in Thailand	Amonhaemanon D		x		
Financial Literacy Confidence and Gambling Intensity among Informal Labor	Amonhaemanon D		x		
Financial stress and gambling motivation: the importance of financial literacy	Amonhaemanon D				x
Financial literacy and its relation to lottery gambling consumption	Cho S		x		
Financial Literacy and Gambling Behavior in the United States	Watanapongvanich S.; Binnagan P.; Putthinun P.; Saidur Rahim Khan M.; Kadoya Y	x			
Characteristics of Chinese Lottery Consumers’ Financial Literacy and its Relationship with Problem Lottery Gambling	Wang Y.; Yang Z.; Xin Z			x	
Financial Literacy and Gambling Behavior: Evidence from Japan	Watanapongvanich S.; Binnagan P.; Putthinun P.; Saidur Rahim Khan M.; Kadoya Y	x			
Gamblers, scratchers and their financial education	Becchetti L.; Bellucci D.; Rossetti F	x			

### Sample Size and Recruitment Methods

The analysis shows (Table 4) that the average sample size across the studies is around 2887 participants. On average, women make up approximately 52.73% of these samples, indicating a fairly balanced representation of genders in the research studies.

**Table 4:** Sample and sample strategy

Title	Sample size	Multi – stage randomization	Multistage sampling	Data from other source	Field sampling	Stratified sampling method combined with quota sampling	Online Recruitment
Financial Literacy and Financial risk tolerance of lottery gamblers in Thailand	N = 995 (70% women)	x					
Financial Literacy Confidence and Gambling Intensity among Informal Labor	N = 995 (w 70% women)	x					
Financial stress and gambling motivation: the importance of financial literacy	N = 995 (w 70%)		x				
Financial literacy and its relation to lottery gambling consumption	500 (W 50%)			x			
Financial Literacy and Gambling Behavior in the United States	4251 (W 56%)						x
Characteristics of Chinese Lottery Consumers’ Financial							
Literacy and its Relationship with Problem Lottery Gambling	N = 316 (W 16,77%.); General population N = 10.058 (W 51,9%)			x	x		
Financial Literacy and Gambling Behavior: Evidence from Japan	N = 3687 (W 50%)			x			
Gamblers, scratchers and their financial education	N = 400 (W 53%)						x

The studies included used a variety of recruitment methods to ensure representative and accurate samples. Some studies employed complex techniques such as multistage randomization and multistage sampling, which divide the population into subgroups and apply random selection processes at each level. These methods are particularly useful for obtaining an accurate representation of the overall population and reducing selection bias. Other studies utilized data from established national surveys, such as the National Financial Capability Study in the United States and the Preference Parameters Study conducted by Osaka University in Japan. The use of data from these broad national surveys increases the generalizability and robustness of the results. Furthermore, some studies employed specific methods such as field sampling for groups of players and stratified sampling combined with quota sampling to obtain representative samples of the general population in China. These approaches aim to ensure that the samples accurately reflect both specific subgroups and the general population. Finally, online recruitment was also used, offering a quick and convenient method for data collection, although it may have limitations in terms of representativeness. Overall, the use of different recruitment and sampling methods in the studies highlights the importance of adopting appropriate strategies to obtain representative samples, reduce bias, and ensure the validity of the results.

### Constructs Analyzed Alongside Financial Literacy in Studies

In summary, the authors also present other economic constructs examined in relation to gambling (Table 5). One of these is the “Risk Tolerance”, that is the level of risk an individual is willing and able to endure in financial decisions (Bodie Kane, & Marcus, 2014). This should be distinguished from the “Perceived Risk Tolerance”, namely how an individual personally perceives their own ability to handle financial risk (Hanna Montalto, & Yuh, 2011). Instead, Financial education (Lusardi, A., & Mitchell, 2014) refers to the process of acquiring knowledge and skills to make informed and effective financial decisions. It encompasses understanding financial concepts such as budgeting, saving, investing, and managing debt, and aims to improve individuals' financial literacy and capability. “Financial education” is the process of learning about financial matters, while financial literacy is the actual knowledge and skills gained from that education. In the studies examined, there are two other important concepts. The “Financial confidence” pertains to how self-assured individuals feel about their financial abilities (Lusardi, A., & Mitchell, O. S. 2014), “Perceived financial literacy” concerns how individuals assess their own financial knowledge (van Rooij M., Lusardi A., & Alessie, R. 2011). The frequency of other economic constructs in the studies was analyzed to determine their prevalence. Both concepts are interrelated but focus on different aspects of financial self-perception. Almost half of the studies we included focused solely on Financial Literacy (Fig. 7). Two studies also addressed Financial Confidence, one study investigated Risk Tolerance, one study explored Perceived Risk Tolerance, and one study covered Financial Education, while another study included both Perceived Risk Tolerance and Financial Education.

**Table 5:** Economic constructs

Title	Financial Literacy	Financial Confidence	Risk Tolerance	Perceived Financial Literacy	Perceived Risk tolerance	Financial Education
Financial Literacy and Financial risk tolerance of lottery gamblers in Thailand	x	x	x			
Financial Literacy Confidence and Gambling Intensity among Informal Labor	x	x				
Financial stress and gambling motivation: the importance of financial literacy	x			x	x	
Financial literacy and its relation to lottery gambling consumption	x					
Financial Literacy and Gambling Behavior in the United States	x					
Characteristics of Chinese Lottery Consumers’ Financial Literacy and its Relationship with Problem Lottery Gambling	x					
Financial Literacy and Gambling Behavior: Evidence from Japan	x					x
Gamblers, scratchers and their financial education	x					

### Methodologies, Controls, and Quantitative Findings

Below, a brief overview of the methodologies and quantitative results of the analyzed studies is provided. The methodologies used by the authors are reported in the table below (Table 6).

**Table 6:** Methodologies used by the authors

	Methodologies	Causal Link
Financial Literacy and Financial Risk Tolerance of Lottery Gamblers in Thailand (Amonhaemanon D.)	Multinomial logistic regression models and MANOVA	No
Financial Literacy Confidence and Gambling Intensity among Informal Labor (Amonhaemanon D.)	ANOVA, and correlation tests	No
Financial Stress and Gambling Motivation: The Importance of Financial Literacy (Amonhaemanon D.)	Logistic regressions	No
Financial literacy and its relation to lottery gambling consumption (Seungyeon C.)	Ordered Probit model with an endogenous regressor	Yes
Financial Literacy and Gambling Behavior in the United States (Watanapongvanich S. et al.)	Probit and Probit-IV regression models	Partially
Characteristics of Chinese Lottery Consumers’ Financial Literacy and its Relationship with Problem Lottery Gambling (Wang Y. et al.)	Propensity Score Matching (PSM), t-tests, and linear regression	No
Financial Literacy and Gambling Behavior: Evidence from Japan (Watanapongvanich S. et al.)	Probit-IV regression model	Yes
Gamblers, scratchers and their financial education (Becchetti L. et al.)	Logit econometric models	No

#### Financial Literacy and Financial Risk Tolerance of Lottery Gamblers in Thailand (Amonhaemanon D.)

The study uses multinomial logistic regression models and multivariate analysis of variance (MANOVA) to examine the relationship between financial literacy (FL) and gambling. Although it is not possible to establish a direct causality, controls for age, gender, education level, income, and psychological factors such as risk tolerance and perceived financial confidence (FLC) are included. The results show that a higher FLC is associated with a higher likelihood of gambling, with a 25% increase in the probability of frequent gambling. In contrast, a higher level of education reduces the risk of gambling. Among pathological gamblers, only 32.33% have a high level of FL, while 67.67% have a low level. Among social gamblers, 48.39% have a high level of FL, highlighting that higher FL is less common among groups with intense gambling problems. However, the model explains only 16.3% of the overall variability, suggesting that additional factors may influence the phenomenon.

#### Financial Literacy Confidence and Gambling Intensity among Informal Labor (Amonhaemanon D.)

The study uses a cross-sectional survey of 995 participants, classified into four groups based on actual (FL) and perceived financial literacy (FLC): Group 1 (high FL, high FLC), Group 2 (low FL, high FLC), Group 3 (high FL, low FLC), and Group 4 (low FL, low FLC). The analysis, conducted using statistical techniques such as analysis of variance (ANOVA) and correlation tests, examines the relationship between financial literacy and gambling intensity.

The analyses include demographic variables (age, gender, income, marital status, education) and behavioral variables (debt and gambling intensity). The results show that an increase of one unit in actual FL reduces the probability of belonging to a high-intensity gambling category by 1.4% (p < 0.05), while an increase of one unit in FLC increases the likelihood of gambling by 12.8% (p < 0.01).

#### Financial Stress and Gambling Motivation: The Importance of Financial Literacy (Amonhaemanon D.)

This study analyzes the influence of financial literacy (FL) on financial stress and gambling motivations using logistic regressions. The analyses include controls and mediators such as demographic variables (age, gender, income, education) and economic variables (debt, savings, net income), as well as risk tolerance and gambling motivations (economic, psychological, social). The results show that higher FL is inversely correlated with both financial stress levels and gambling intensity, although the causal relationship between gambling and FL remains uncertain. Specifically, participants who perceived higher FL were less likely to experience financial stress (Exp(B) = 0.689, p < 0.05).

#### Financial literacy and its relation to lottery gambling consumption, (Seungyeon C.)

The study analyzes the relationship between financial literacy (FL) and lottery consumption, using an Ordered Probit model with an endogenous regressor to establish a causal link. FL is treated as a potentially endogenous variable with respect to gambling frequency, using the average percentage of college graduates by geographic area (ZCTA) as an instrumental variable. The validity of the instrument is confirmed by its relevance (coefficient = 0.488, t-statistic = 5.66, p < 0.0001) and the satisfaction of the exogeneity condition, according to which educational level influences lottery consumption only through FL.

The results show that an increase of one standard deviation in FL increases the probability of never gambling by 36.1% and reduces the probability of gambling less than once a month by 11.5%. The model includes several control variables, such as age, gender, income, educational level, and risk attitude, although it acknowledges a potential bias from unobservable factors.

#### Financial Literacy and Gambling Behavior in the United States, (Watanapongvanich S.; Binnagan P.; Putthinun P.; Saidur Rahim Khan M.; Kadoya Y.)

The study explores the relationship between financial literacy (FL) and gambling behavior in the United States, using probit and probit IV regression models to address the issue of endogeneity. The initial results (models 1.1 and 1.2) show no significant relationship between FL and gambling frequency, with coefficients of -0.0925 and -1.035, respectively. Similarly, in models that include the variable of access to gambling through electronic gaming machines (EGM), no significant correlation is found (models 2.1 and 2.2). However, when analyzing states with and without EGMs separately, differences emerge. In states without EGMs, financial literacy has a significant impact, reducing the frequency of gambling (model 3.2: coefficient -3.189, p < 0.1). In states with EGMs, the effect of FL is negligible, with a coefficient of -0.577 in the IV probit model (4.2).

#### Characteristics of Chinese Lottery Consumers’ Financial Literacy and its Relationship with Problem Lottery Gambling, (Wang Y.; Yang Z.; Xin Z.)

The study uses Propensity Score Matching (PSM) to reduce the differences between a sample of 316 lottery consumers and a reference population of 10,058 people, balancing sociodemographic factors. T-tests showed that lottery consumers have significantly lower scores in all areas of financial literacy compared to the general population (with average differences ranging from 0.32 to 0.69, indicating medium-sized effects). The Homo Sociologicus index (which includes financial ethics and wealth values) shows a marked difference (d = -0.70), suggesting a significant correlation with problematic gambling behavior. Additionally, a linear regression was conducted to explore how different dimensions of financial literacy influence problematic gambling. The results indicate that financial knowledge and financial capability are not significantly related to problematic gambling (p > 0.05), while financial ethics (β = -0.23, p < 0.001) and wealth values (β = -0.19, p = 0.002) are significant protective factors, reducing the likelihood of problematic gambling.

#### Financial Literacy and Gambling Behavior: Evidence from Japan, (Watanapongvanich S.; Binnagan P.; Putthinun P.; Saidur Rahim Khan M.; Kadoya Y.)

The main variables analyzed in the study are financial literacy and financial education, the latter measured through a retrospective question about the financial education received during childhood. To address the issue of endogeneity, the study uses the father's education level as an instrumental variable, which is correlated with financial literacy but not with gambling behavior. The results obtained from a probit-instrumental variable (IV) model show a significant negative relationship between financial literacy and gambling frequency: higher financial literacy is associated with a lower probability of frequent gambling, with significant coefficients of −2.344 (p < 0.01), −2.336 (p < 0.01), and −2.265 (p < 0.05) in the various models analyzed.

#### Gamblers, scratchers and their financial education, (Becchetti L.; Bellucci D.; Rossetti F.)

The data were analyzed using logit econometric models to estimate the probability of participating in gambling based on variables such as financial literacy test results, socio-demographic characteristics, and financial preferences. The results indicate that slot/videopoker players and scratch card consumers have lower financial literacy compared to non-gamblers. The models suggest that one additional correct answer on financial literacy questions reduces the probability of participating in gambling by 7-8%, and for scratch card consumers, a better understanding of risk diversification reduces this probability by 10%. The analysis highlights a negative correlation between low financial literacy and a higher propensity to gamble, although no direct causal link can be established.

### Results and Implications

#### Financial Literacy and Gambling Frequency

Studies indicate a negative relationship between financial literacy and the frequency of lottery ticket purchases. In Thailand, higher financial literacy is associated with a lower frequency of lottery ticket purchases. However, increased confidence in financial literacy and risk tolerance can lead to more frequent gambling behaviors.

In the U.S. (Watanapongvanich S. et al. 2022), financial literacy is not significantly correlated with gambling frequency at the national level but becomes significant in states with easy access to electronic gaming machines. This suggests that easier access to gambling may amplify gambling effects, making financial literacy a more effective protective factor in contexts with less access.

Lottery consumers in China exhibit significantly lower levels of financial literacy compared to the population. However, only financial ethics and wealth values are negatively correlated with problematic gambling, indicating that some dimensions of financial literacy may have a more direct protective impact.

Therefore, in China and the United States, results are less clear, with some analyses showing no significant correlation between financial literacy and gambling frequency. This suggests that local and context-specific factors may influence the effectiveness of financial literacy as a protective measure.

The study by Becchetti et al. (2018) revealed that scratch card and slot/video poker players generally have lower financial literacy compared to non-players. Specifically, only 27% of scratch card players and 24% of slot/video poker players answered all three financial literacy questions correctly, compared to 37% among non-players and 49% among those who avoid scratch cards for economic reasons. The percentages of correct answers are significantly higher among non-players and those who avoid gambling for economic reasons. Respondents with good financial literacy are 7–8% less likely to engage in slot/video poker gambling.

In six of the eight included studies, the authors noted limitations related to the measurement scales used. This underscores the need to consider more detailed and structured measurement methods to investigate the relationship between financial literacy and problematic gambling. It would also be useful for future research to investigate more deeply the role of “Perceived financial literacy” or “Financial Literacy Confidence”, as, from the studies reviewed, its relationship with problematic gambling does not appear to be very clear.

## Discussion

Pathological gambling is a growing phenomenon with devastating impacts on individuals and families. While research on risk factors is well-developed, there is a need to deepen the understanding of protective factors. This systematic analysis of the literature explores whether financial literacy and economic attitudes can serve as protective factors against pathological gambling, with a particular focus on young people.

The studies included in the review cover the period from 2018 to 2023, highlighting that the topic is relatively recent. This allows us to understand that the relationship between financial literacy and gambling is still a relatively underexplored aspect that has been gaining increasing interest in recent years. This is likely due first to the expansion of online gambling and the accessibility of various forms of betting, which has led to a rise in problematic gambling cases, consequently drawing researchers' attention. Second, it is also due to the recognition of the growing importance of financial literacy in recent years as a highly useful tool for achieving lasting financial well-being (Lusardi, A. & Mitchell, 2014). Additionally, recent efforts by both governmental and non-governmental organizations to improve financial education have created greater research interest in this field (OECD, 2015). The geographical distribution is varied: most studies come from Thailand, followed by the United States, Italy, China, and Japan. This suggests the influence of different cultural and socioeconomic contexts on the results and offers diverse perspectives on the role of financial literacy.

The measurement of problematic gambling behavior is primarily based on frequency, but some studies use more complex tools, such as the Li et al. (2012) scale, which explores dimensions like harmful behavior and compulsive disorder. Problematic gambling is a complex phenomenon encompassing both behavioral and psychological elements. This includes behavioral aspects such as comorbidities often associated with it, like excessive alcohol consumption or smoking (Petry, N. M., Stinson, F. S., & Grant, B. F., 2005), and psychological aspects such as dysphoric moods (Blaszczynski, A., & Nower, L., 2002). Additionally, motivations driving individuals to compulsive gambling behavior (Thomas, A. C. et al., 2011) and the consequences of such behavior (Dowling, N. A. et al., 2021) are essential factors to consider for a clearer and more detailed understanding of gambling problems. These are just a few of the aspects that should be examined when assessing gamblers. Doing so is crucial not only for accurately identifying problematic situations, which may be connected to or exacerbated by these factors, but also for gaining vital insights into the psychological and behavioral profiles of individuals who develop compulsive gambling habits. This would enable a more precise understanding of the relationship between problematic gambling, with its complex and multifaceted components, and financial literacy.

The assessment of financial literacy is primarily guided by the Lusardi and Mitchell method, which evaluates understanding of compound interest, inflation, and risk diversification. However, alternative approaches, such as those developed by Xin et al. (2020), offer a more comprehensive and integrated perspective on financial literacy. It is crucial to consider that, according to the OECD Recommendation on Financial Literacy (2020), awareness, skills, attitudes, and behaviors are essential dimensions of financial literacy. When analyzing financial literacy, it is important to account for its multidimensional nature to examine other aspects such as financial behaviors and attitudes. Therefore, it is essential for future research to employ precise and multidimensional methods to measure both problematic gambling behavior and financial literacy, to gain a more thorough and accurate understanding of their interrelationships. Another crucial aspect that emerges is the need to establish clearer causal links between financial literacy and problematic gambling. Most studies analyze correlations but fail to provide sufficient evidence to establish a direct causal relationship. To address this gap, future research could adopt longitudinal designs that track how changes in financial literacy levels influence gambling behavior over time. Additionally, it would be beneficial to integrate experimental or quasi-experimental approaches, such as randomized controlled trials, to examine the direct effects of financial education interventions on problematic gambling behaviors.

## Conclusions

The review suggests that financial literacy may play a protective role against pathological gambling, but with significant variations based on cultural context and the level of gambling accessibility. It is essential to develop targeted financial education programs, particularly in contexts with high availability of gambling opportunities, and to consider the multidimensional aspects of financial literacy for more effective protection. Financial education programs should be designed with cultural specifics and the level of gambling access in mind to maximize their preventive impact.

Ongoing research is needed to explore how cultural and socioeconomic variables influence the effectiveness of financial literacy as a protective factor. Additionally, longitudinal studies could help better understand how changes in financial literacy levels affect gambling behavior over the long term. Using more accurate and integrated measurement methods is crucial for obtaining more precise results.

Investing in financial education could represent an effective strategy to reduce pathological gambling behaviors, particularly among youth and in areas with high gambling availability. This approach could contribute to more targeted prevention and increased awareness of the risks associated with gambling.

## Data Availability

The data that support the findings of this study are available from the corresponding author, upon reasonable request.
